# Temporal trends of ischemic stroke attributable to diet high in sodium in China from the global burden of disease study 2021

**DOI:** 10.3389/fnut.2025.1513981

**Published:** 2025-03-13

**Authors:** Jiaming Cui, Zhiwei Xu, Yang Dai, Qi Wang, Zhihui Hou, Yongchen Zhang, Hongling Jia

**Affiliations:** ^1^School of Acupuncture and Tuina, Shandong University of Traditional Chinese Medicine, Jinan, China; ^2^Department of Acupuncture, Second Affiliated Hospital of Shandong University of Traditional Chinese Medicine, Jinan, China; ^3^Shandong University of Traditional Chinese Medicine, Jinan, China; ^4^Department of Acupuncture, Affiliated Hospital of Shandong University of Traditional Chinese Medicine, Jinan, China

**Keywords:** diet high in sodium, ischemic stroke, disease burden, GBD 2021, China, joinpoint regression, age-period-cohort analysis

## Abstract

**Background:**

Ischemic stroke is a significant global health burden, with high sodium intake recognized as a key risk factor. This study aimed to assess the disease burden of ischemic stroke attributable to diet high in sodium in China from 1990 to 2021. Additionally, we analyzed the influence of age, period, and cohort effects on the trends in ischemic stroke burden and projected the disease burden from 2022 to 2036.

**Methods:**

Data from the Global Burden of Disease Study 2021 (GBD 2021) were used to analyze the ischemic stroke burden among high-risk populations in China. Annual average percent change (AAPC) was calculated using Joinpoint regression models to evaluate trends in ischemic stroke burden from 1990 to 2021. Age-period-cohort models were employed to estimate the independent effects of age, period, and cohort on the ischemic stroke burden, and to project the burden from 2022 to 2036 using Bayesian age-period-cohort models.

**Results:**

From 1990 to 2021, ischemic stroke mortality attributable to diet high in sodium in China showed a continuous increase, while the age-standardized mortality and disability-adjusted life years (DALYs) significantly declined. In the age-period-cohort analysis, the age effect on ischemic stroke burden increased steadily over the study period. Period effects revealed an initial decline in the relative risk (RR) of ischemic stroke mortality and DALY rates, followed by an increase in cohorts born before 2004–2009, and a gradual decline in cohorts born after that period. Cohort effects demonstrated a continuous decline in the relative risk of ischemic stroke mortality and DALY rates from 1990 to 2021.

**Conclusion:**

This study found that ischemic stroke attributable to a diet high in sodium in China fluctuated from 1990 to 2021, with a declining trend observed in recent years. Projections indicate that this downward trend will continue. Age and birth period are key factors influencing the disease burden, with older adults and men being particularly affected. Future policy efforts should focus on enhancing health management in high-risk populations to further reduce the burden of ischemic stroke linked to high sodium intake.

## Introduction

Stroke is a leading cause of death and disability globally, imposing a substantial burden on individuals and society (1, 2). Ischemic stroke, caused by occlusion of cerebral arteries, accounts for approximately 87% of all stroke cases and represents the majority of stroke-related morbidity (3). China has the highest estimated risk of stroke worldwide, with this risk continuing to rise (4). In 2019, China reported 2.19 million stroke-related deaths and 45.9 million disability-adjusted life years (DALYs), with ischemic stroke responsible for 1.03 million deaths, or about 47% of total stroke mortality (5). A cross-sectional study revealed that by 2020, more than 80% of stroke cases in China were ischemic strokes (6). Consequently, ischemic stroke presents a major public health challenge in China, making it essential to assess temporal trends in its burden to guide interventions and improve public health outcomes.

Previous studies from the Global Burden of Disease (GBD) collaborators have shown that most stroke-related disease burden is attributable to key environmental and lifestyle risk factors (7). The Global Burden of Disease Study 2019 (GBD 2019) identified diet high in sodium (DHIS) as a significant risk factor for stroke, contributing to a marked increase in stroke deaths worldwide (8). Despite this, the stroke burden associated with DHIS has not received sufficient attention (7). DHIS is linked to hypertension, cardiovascular disease, and other chronic conditions (9–11), and is a particularly important risk factor for ischemic stroke in China compared to other countries (12). However, the prevalence and trends of ischemic stroke caused by DHIS in China remain underexplored, and the epidemiological characteristics and patterns of this burden are not well understood. To address this gap, we used data from the Global Burden of Disease Study 2021 (GBD 2021) to systematically estimate and predict trends in ischemic stroke burden related to DHIS in China from 1990 to 2036.

## Methods

### Data resource

Data for this study were obtained from the Global Burden of Disease Study 2021 (GBD 2021),^1^ which was conducted by the Institute for Health Metrics and Evaluation (IHME) to provide a comprehensive assessment of ischemic stroke burden and its associated risk factors over a 32-year period (1990–2021). GBD 2021 offers a comparative evaluation of 371 diseases and injuries, along with 88 risk factors, across 204 countries and territories in 21 regions and 7 super regions (2, 13). For this study, we extracted data on the burden of ischemic stroke attributable to a diet high in sodium (DHIS) in China. Ischemic stroke was defined according to the 10th edition of the International Classification of Diseases (ICD-10) code (I63.0–I63.9), and DHIS was defined as 24-h mean urinary sodium excretion exceeding the theoretical minimum risk exposure level (TMREL) of 1–5 g/day (13). The data were stratified into 15 age groups (25–29, 30–34..., 90–94, and 95+), and age-standardized rates were calculated using the GBD 2021 standard population. We assessed the number of deaths, disability-adjusted life years (DALYs), years of life lost (YLL), and years lived with disability (YLD), alongside age-standardized mortality rates (ASMR), DALYs, YLLs, and YLDs, to quantify the burden of ischemic stroke linked to DHIS in China.

### Joinpoint regression analysis

The Joinpoint regression model, a series of linear statistical models, was used to analyze the temporal trends in ischemic stroke burden attributable to DHIS in China (14). This model applies the least squares method to estimate changes in incidence rates, thereby avoiding the subjectivity inherent in typical linear trend analyses. By calculating the sum of squared residuals between estimated and actual values, the model identifies inflection points and divides the overall trend into sub-segments. Each segment trend is individually assessed, and the overall trend is evaluated using the Annual Percent Change (APC) for each segment and the Annual Average Percent Change (AAPC) for the entire trend. The method offers clear segmentation of trends and utilizes Monte Carlo permutation tests to identify the critical parameters of the joinpoints. Bonferroni correction is applied to preserve the overall significance level, thereby facilitating the interpretation of key transitions in the data (14). In contrast, spline regression analysis, although more widely used, fits nonlinear relationships by constructing smooth curves between data points. While spline regression offers greater flexibility in fitting, it typically fails to detect distinct turning points in trends and is thus better suited for data with smoother fluctuations. Within the framework of mixed models, Joinpoint LR analysis effectively captures and visually displays trend changes at different stages, whereas spline regression provides a smooth nonlinear fit across the entire dataset. In this study, we utilized Joinpoint software (version 4.9.1.0; National Cancer Institute, Rockville, MD, United States) to calculate the temporal trends in ischemic stroke burden due to DHIS in China from 1990 to 2021. We also computed the AAPC and compared it to zero, with an AAPC greater than zero indicating an upward trend and an AAPC less than zero indicating a downward trend. A *p*-value of less than 0.05 was considered statistically significant.

### Age-period-cohort analysis and projection

We employed an age-period-cohort (APC) model to evaluate the impact of DHIS on ischemic stroke burden. Unlike traditional linear models, APC models simultaneously decompose trends by age, period, and birth cohort, providing more robust estimates (15, 16). Age effects describe changes across the life course, period effects capture societal trends or events affecting populations at a given time, and cohort effects highlight the influence of specific birth cohorts on disease burden (17). Mortality and disability-adjusted life years (DALY) data for ischemic stroke caused by DHIS in China were organized by 5-year age groups and analyzed using the APC model analysis toolkit developed by the Institute for Health Metrics and Evaluation (IHME).^2^ In this model, net drift reflects the overall log-linear trend across periods and cohorts, indicating the annual percentage change, while local drift represents log-linear trends by age group, reflecting the annual percentage changes within each age group. Longitudinal age curves show fitted age-specific ratios for the reference cohort, adjusted for period bias. The cohort or period relative risk (RR) represents the cohort or period risk relative to the reference cohort or period, adjusted for age and nonlinear period or cohort effects (18, 19). The estimable function was tested using the Wald *c*^2^ test, and statistical analysis was performed using two-sided tests with an alpha level of 0.05.

To predict the ischemic stroke burden due to DHIS in China from 2022 to 2036, we applied a Bayesian age-period-cohort (BAPC) model using integrated nested Laplace approximation (INLA). The BAPC model effectively captures the interaction effects between age, age-cohort, and cohort, offering a significant improvement over traditional APC models by addressing the linear dependencies among these variables (20). Additionally, INLA provides efficient Bayesian inference, making it particularly well-suited for large-scale datasets. INLA offers advantages in computational speed, interpretability, model transparency, and overall efficiency. In contrast, other computational models, such as generalized additive models (GAM), while capable of modeling nonlinear relationships, are less adept at handling complex time and interaction effects compared to the BAPC model. Although machine learning techniques may excel in predictive accuracy, their “black box” nature hinders interpretability, making them less suitable for epidemiological studies that require clear, explicit explanations. BAPC modeling was conducted using the nordpred, INLA, and BAPC packages in R (version 4.3.2).

## Result

### Temporal trend in the burden of ischemic stroke attributable to DHIS by sex in China

Table 1 presents the ischemic stroke disease burden attributable to DHIS in China from 1990 to 2021. During this period, the number of ischemic stroke deaths due to DHIS increased significantly, from 76,328 in 1990 to 173,363 in 2021. However, the age-standardized mortality rate (ASMR) declined from 11.43 per 100,000 to 8.77 per 100,000 (AAPC = −0.88, *p* < 0.001). Similarly, the age-standardized rate of DALYs (ASDR) decreased from 241.79 per 100,000 to 190.04 per 100,000 (AAPC = −0.80, *p* < 0.001). The age-standardized rates for YLDs increased from 25.58 per 100,000 to 32.92 per 100,000, while the rates for YLLs decreased from 216.21 per 100,000 to 157.12 per 100,000 over the same period. Both ASMR and ASDR exhibited statistically significant changes, showing a pattern of initial decline, followed by an increase, and then another decline between 1990 and 2021 (Supplementary Table 1). In terms of sex-specific data, both the absolute numbers and age-standardized rates indicate that men bear a higher disease burden than women across all metrics.

**Table 1:** Analysis of ischemic stroke deaths, DALYs, YLDs, YLLs and ASRs associated with high sodium intake in China, 1990–2019.

	1990		2021		AAPC	*t*	*p*
	Number (95% UI)	ASR, per 100,000 (95% UI)	Number (95% UI)	ASR, per 100,000 (95% UI)			
Both							
Deaths	76,328 (25,342,140,977)	11.43 (3.25,22.08)	173,363 (46,361,351,905)	8.77 (2.17,18.17)	−0.88	−6.17	<0.001
DALYs	1,902,567 (708,070,3,422,739)	241.79 (81.56,441.27)	4,027,007 (1,335,587,7,834,878)	190.04 (60.47,374.29)	−0.80	−6.56	<0.001
YLDs	220,601 (79,009,406,765)	25.58 (9.00,47.73)	713,152 (230,786,1,328,424)	32.92 (10.41,61.78)	0.79	12.83	<0.001
YLLs	1,681,966 (629,045,3,037,598)	216.21 (72.39,398.61)	3,313,855 (1,050,958,6,415,218)	157.12 (47.33,309)	−1.06	−8.67	<0.001
Male							
Deaths	45,249 (16,971,82,863)	15.31 (4.83,28.85)	113,577 (34,025,227,502)	13.04 (3.46,27.09)	−0.53	−2.83	0.005
DALYs	1,140,172 (456,013,2,034,263)	310.66 (112.11,564.46)	2,631,614 (922,735,4,986,307)	265.88 (87.37,516.66)	−0.52	−3.78	<0.001
YLDs	115,871 (47,061,208,439)	28.66 (10.85,52.43)	397,632 (144,987,717,282)	37.79 (13.36,68.47)	0.89	25.76	<0.001
YLLs	1,024,301 (407,364,1,860,974)	282.00 (100.90,519.55)	2,233,982 (767,249,4,321,952)	228.09 (72.39,451.29)	−0.70	−4.75	<0.001
Female							
Deaths	31,079 (7,985,60,265)	8.67 (1.92,17.34)	59,786 (11,918,136,387)	5.56 (1.05,12.8)	−1.41	−9.25	<0.001
DALYs	762,394 (237,130,1,436,027)	186.17 (52.78,354.91)	1,395,393 (370,240,2,957,823)	125.58 (32.57,267.81)	−1.28	−10.37	<0.001
YLDs	104,730 (33,662,203,947)	23.44 (7.31,46.09)	315,519 (87,521,621,691)	28.45 (7.76,56.54)	0.60	23.02	<0.001
YLLs	657,664 (198,101,1,256,432)	162.73 (44.88,317.69)	1,079,873 (266,188,2,360,822)	97.13 (23.29,213.55)	−1.65	−11.53	<0.001

ASR, age-standardized rate; DALYs, disability-adjusted life years; YLDs, years of life lived with disability; YLLs, years of life lost; UI, uncertainty interval.

### The burden of ischemic stroke attributable to DHIS across different age groups in China

**Figure 1:**
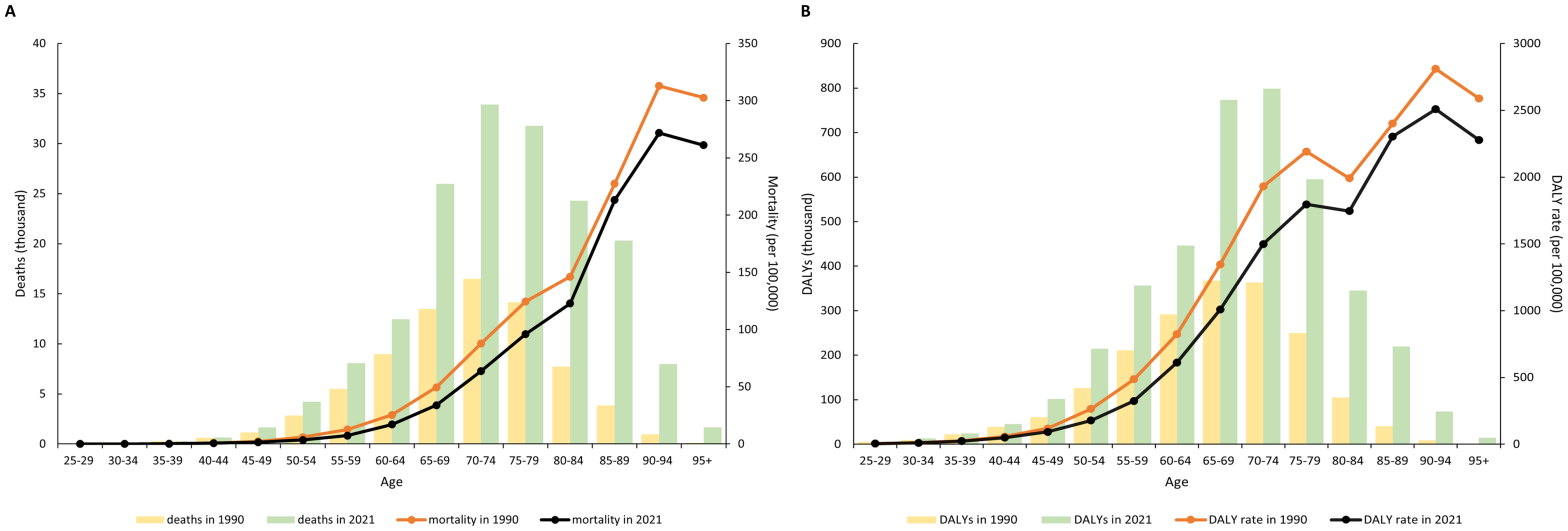
Burden of ischemic stroke attributable to diet high in sodium in China, by age group, 1990 and 2021. **(A)** mortality (per 100,000) and deaths (thousand); **(B)** DALY rate (per 100,000) and DALYs (thousand).

Figure 1 illustrates the burden of ischemic stroke attributable to DHIS across different age groups in China for the years 1990 and 2021. Overall, the burden of ischemic stroke is predominantly observed in individuals aged 65 years and older. Compared to 1990, there was a significant increase in the number of deaths and DALYs in 2021, particularly among the elderly population. However, this trend was not reflected in the mortality and DALY rates, as both rates for all age groups in 2021 were lower than those in 1990. YLL, YLD, and age-standardized YLLs exhibited similar patterns, while age-standardized YLDs ratios for all age groups were higher in 2021 than in 1990 (Supplementary Figure 1).

### Age, period and cohort effects on the burden of ischemic stroke between 1990 to 2021

**Figure 2:**
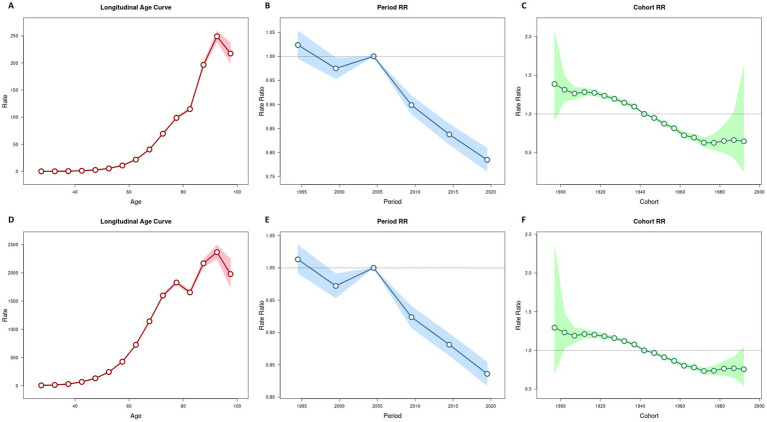
Age–period–cohort analysis for death and DALY rates of ischemic stroke attributable to diet high in sodium in China. **(A)** Age efect for death rate. **(B)** Period efect for death rate. **(C)** Cohort efect for death rate. **(D)** Age efect for DALY rate. **(E)** Period efect for DALY rate. **(F)** Cohort efect for DALY rate.

Table 2 presents the net drift values for ischemic stroke attributable to DHIS in China, indicating a decrease of −1.076% (95% CI -1.257 to −0.895) per year for mortality and − 0.760% (95% CI -0.870 to −0.650) per year for DALYs. After controlling for period and cohort effects, we observed that the mortality rate for ischemic stroke caused by DHIS increased with age, and the DALY rate exhibited a similar upward trend with advancing age. Regarding the period effect, the RR for ischemic stroke mortality and DALY rates initially decreased before increasing in cohorts born prior to 2004–2009; this RR subsequently showed a gradual decline with later years of birth in cohorts born after 2004–2009. The cohort RR for ischemic stroke mortality and DALYs decreased consistently from 1990 to 2021 (Figure 2). Changes in mortality and DALY rates for men and women, stratified by age, period, and cohort, are detailed in Supplementary Tables 2, 3, as well as Supplementary Figures 2, 3.

**Table 2:** Parameter estimates of age, period, and cohort effects on burden of ischemic stroke attributable to diet high in sodium in China.

Type	Net drift (% per years; 95% CI)	*p* value		
		All local drifts = net drift	All cohort deviations = 0	All period deviations = 0
Death	−1.076 (−1.257 to-0.895)	<0.001	<0.001	<0.001
DALY	−0.760 (−0.870 to-0.650)	<0.001	<0.001	<0.001

DALY, disability-adjusted life year.

### The projected burden of ischemic stroke attributable to DHIS, 2020–2030

**Figure 3:**
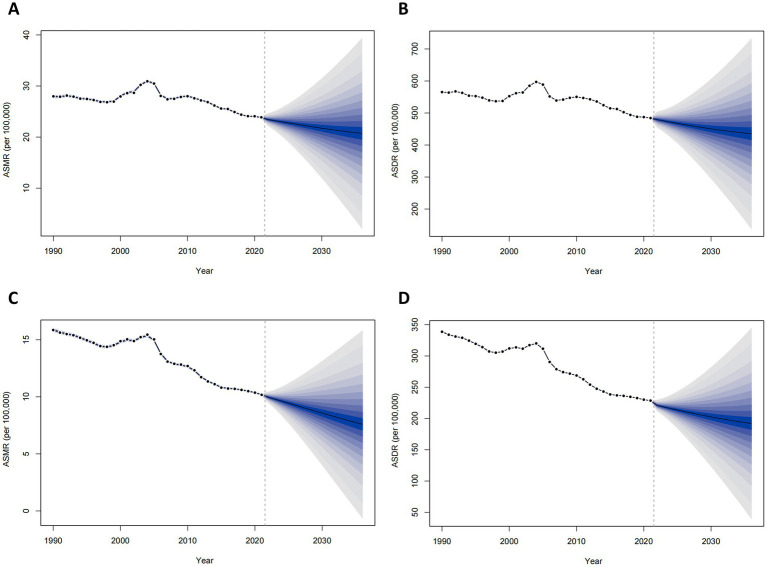
Projected burden of ischemic stroke attributable to diet high in sodium in China, 2022–2036. **(A)** age-standardized mortality rate among males; **(B)** age-standardized DALYs rate among males; **(C)** age-standardized mortality rate among females; **(D)** age-standardized DALYs rate among females.

Projections indicate that ASMR and ASDR for ischemic stroke attributable to DHIS in China will exhibit similar and sustained downward trends for both males and females from 2022 to 2036, as illustrated in Figure 3. Specifically, the ASMR for males is expected to decline from 23.42 per 100,000 in 2022 to 20.73 per 100,000, while the ASDR is projected to decrease from 479.17 per 100,000 to 435.25 per 100,000. For females, the ASMR is anticipated to decrease from 9.96 per 100,000 in 2022 to 7.59 per 100,000, and the ASDR is expected to decline from 220.83 per 100,000 to 192.03 per 100,000. Detailed projections can be found in Supplementary Table 4.

## Discussion

In 2021, stroke ranked as the third leading cause of death and the fourth leading cause of DALYs among non-communicable diseases (NCDs) globally, accounting for 7.3 million deaths and 160.5 million DALYs (7). Ischemic stroke constitutes the majority of all stroke cases, and despite substantial progress in prevention, diagnosis, and treatment, the global burden of ischemic stroke remains significant (5). From 1990 to 2021, the absolute number of deaths and DALYs attributable to ischemic stroke increased substantially (21). Beyond population growth and aging, the rising global burden of ischemic stroke may be linked to heightened exposure to key risk factors, including high body mass index, tobacco use, particulate matter air pollution, excessive alcohol consumption, physical inactivity, renal dysfunction, and DHIS (13, 21, 22). This study provides a comprehensive analysis of the long-term burden of ischemic stroke attributable to DHIS in China from 1990 to 2021, with an assessment of its association with age and sex. We observed a significant increase in DHIS-related ischemic stroke deaths in 2021 compared to 1990. However, ASMR, ASDR, and age-normalized YLL decreased, while age-normalized YLD increased. Notably, the burden of DHIS-related ischemic stroke escalates markedly with age, particularly after the age of 65. Although the overall disease burden has declined in the past decade, the reduction has been more pronounced in women than in men, with the burden in men plateauing in recent years. Furthermore, the disease burden remains higher in men than in women. BAPC prediction models indicate that the disease burden will continue to decline in both sexes over the next 15 years.

Sodium is an essential nutrient for normal physiological function and overall health, necessitating a balanced and adequate intake, as with other key electrolytes (23). The World Health Organization (WHO) recommends a sodium intake of less than 2 grams per day for adults, equivalent to less than 5 grams of salt (24). However, global estimates suggest that the average daily sodium intake among adults ranges from 3 to 6 grams, exceeding recommended levels (25). Numerous studies have established that reducing sodium intake is an effective strategy for controlling hypertension and lowering the burden of cardiovascular disease (26–29). Research in the United States has demonstrated that reduced salt consumption significantly lowers blood pressure, subsequently decreasing the risk of coronary heart disease and stroke (30). Furthermore, low-sodium salt substitutes have been shown to produce significant reductions in both systolic and diastolic blood pressure (31). A cohort study in China similarly found that reduced salt intake was associated with a marked reduction in stroke risk (32). Thus, the blood pressure-lowering effect of reduced sodium intake likely contributes to the decreasing disease burden of ischemic stroke. By 2018, sodium intake in the Chinese population had decreased from 16 grams per day in 1991 to approximately 11 grams per day, a decline that may partly explain the observed reduction in the disease burden of ischemic stroke due to DHIS in China (33, 34). Additionally, increased public awareness of stroke, improvements in healthcare infrastructure, enhanced emergency medical services, and prevention of stroke risk factors have also contributed to the overall decline in DHIS-related ischemic stroke burden (8).

Our findings indicate that older adults experience the highest mortality and disease burden from ischemic stroke attributable to DHIS, suggesting that aging itself may be a significant risk factor. Previous research has consistently shown that older adults not only have a higher incidence of ischemic stroke but also face poorer functional recovery and higher mortality compared to younger individuals (35). Several factors may contribute to this elevated risk. First, older adults tend to consume more sodium than younger populations. A study in the United States indicated that sodium intake recommendations based on absolute values may overestimate adherence among older adults, leading to sodium overload (36). Similarly, many elderly individuals in China are unaware of their excessive sodium intake, often consuming well above the recommended levels (37). In 2009, data from China indicated a relatively high sodium-to-potassium ratio among individuals aged 50 years and older, reflecting higher sodium consumption (38). Second, age-related declines in physical function may increase the risk of ischemic stroke mortality. Immunosenescence—age-associated changes in the immune system—leads to impaired immune responses, exacerbating the risk of stroke-related death (39). Existing studies have demonstrated that over time, immunosenescence amplifies neuroinflammation, gradually altering and deteriorating the immune system, ultimately leading to adverse outcomes in older adults, such as ischemic stroke (40). In humans, weakened immune systems can influence the progression of atherosclerosis, promote vascular stiffness, and contribute to vascular aging, all of which elevate the risk of ischemic stroke (41). Finally, the combination of advanced age and pre-existing comorbidities may further heighten the risk of ischemic stroke mortality (42, 43).

Our study also found that the disease burden of ischemic stroke attributable to DHIS is generally higher in men than in women in China, suggesting that men are at greater risk for ischemic stroke-related disability and death, while women may have improved survival outcomes (8). This disparity may be partially explained by lower sodium intake among women in China compared to men and by the greater sensitivity of women’s blood pressure to reductions in salt intake (44, 45). Additionally, sex hormones play a critical role in stroke outcomes. Growing evidence indicates that estrogens, particularly 17β-estradiol, exert neuroprotective effects following ischemic stroke (46). This protective action is mediated through several mechanisms, including modulation of both local and systemic immune responses after stroke (47). Experimental studies have consistently shown that estrogen acts as a potent immunomodulator and neuroprotective agent in ischemic stroke (48, 49). Lifestyle differences between men and women may also contribute to the disparity in disease burden. Hypertension, the most prevalent risk factor for stroke, tends to be more pronounced in men, with studies showing that men generally have higher blood pressure than women of the same age (50). Moreover, alcohol consumption, which exhibits a J-shaped relationship with stroke risk and mortality, is typically higher in men (51–53). In contrast, women in China tend to lead healthier lifestyles compared to men. These factors may explain the greater reduction in ischemic stroke burden observed among women compared to men over the past decade.

Despite China’s sustained efforts in the treatment and prevention of ischemic stroke over the past decade, the epidemic remains uncontrolled. However, BAPC models suggest that the burden of ischemic stroke attributable to DHIS will continue to decline over the next 15 years, reflecting the partial success of China’s salt reduction initiatives. Research indicates that reducing sodium intake is a cost-effective strategy for lowering cardiovascular disease prevalence and alleviating economic burden (26). Nevertheless, recent studies reveal that the average sodium intake among Chinese adults remains more than twice the recommended upper limit (44). Therefore, further efforts to implement and strengthen sodium reduction policies are necessary to achieve World Health Organization (WHO) targets and to further reduce the burden of ischemic stroke.

While the GBD 2021 study employs rigorous algorithms for data estimation, several limitations exist. First, GBD 2021 relies on modeled data rather than direct observations, which may introduce systematic biases and affect the accuracy and reliability of our findings. Second, the GBD 2021 employs the TMREL to assess the impact of various risk factors. However, China’s vast geographic expanse and considerable economic and cultural diversity may limit the generalizability of TMREL. Dietary patterns in China vary markedly across regions; for instance, coastal areas in the east tend to have higher salt intake, while inland and western regions use different condiments, which could influence dietary habits. Furthermore, economic and cultural differences also shape dietary choices, with urban populations more likely to consume processed foods high in sodium, while rural populations generally adhere to more traditional, less processed diets. Consequently, TMREL may not accurately reflect the exposure risks in low-income or rural populations. Given China’s complex demographic structure and diverse dietary habits, it is crucial to consider these regional and socioeconomic differences when applying TMREL to inform public health policies tailored to specific populations. Third, this study analyzed data at the national level, without considering provincial or rural–urban differences. The use of more granular data could provide insight into specific regional disparities. Finally, BAPC analysis does not account for potential changes in interventions or environmental factors, which may introduce biases in the projections, and thus, the interpretation of these results requires further multi-faceted analyses.

## Conclusion

In conclusion, we observed a substantial increase in DHIS-related deaths and DALYs in China from 1990 to 2021, despite a decline in ASMR and ASDR. The disease burden of ischemic stroke attributable to DHIS was higher among men than women and greater in older adults compared to younger individuals. Although the overall burden of DHIS-related ischemic stroke is decreasing in China, further efforts to control sodium intake and strengthen salt reduction policies are still required. Future initiatives should focus on reducing the burden of ischemic stroke caused by DHIS through more comprehensive and targeted interventions.

## Data Availability

The original contributions presented in the study are included in the article/Supplementary material, further inquiries can be directed to the corresponding author.

## Footnotes

^1^ https://www.healthdata.org

^2^ http://analystools.cancer.gov/apc/
