# Supplementary material for: Temporal trends of ischemic stroke attributable to diet high in sodium in China from the global burden of disease study 2021
Source: Front Nutr. 2025 Mar 13;12:1513981. doi: 10.3389/fnut.2025.1513981 (PMC11966442; doi:10.3389/fnut.2025.1513981)
Supplement: Supplementary file 2 [file Image_1.pdf]

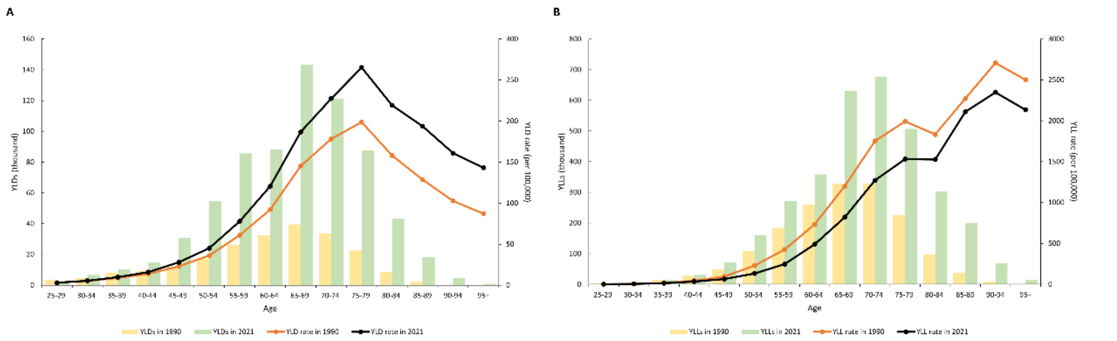

Supplementary FIGURE 1

Burden of ischemic stroke attributable to diet high in sodium in China, by age group, 1990 and 2021. (A) YLD rate (per 100,000) and YLDs(thousand); (B): YLL rate (per 100,000) and YLLs (thousand).

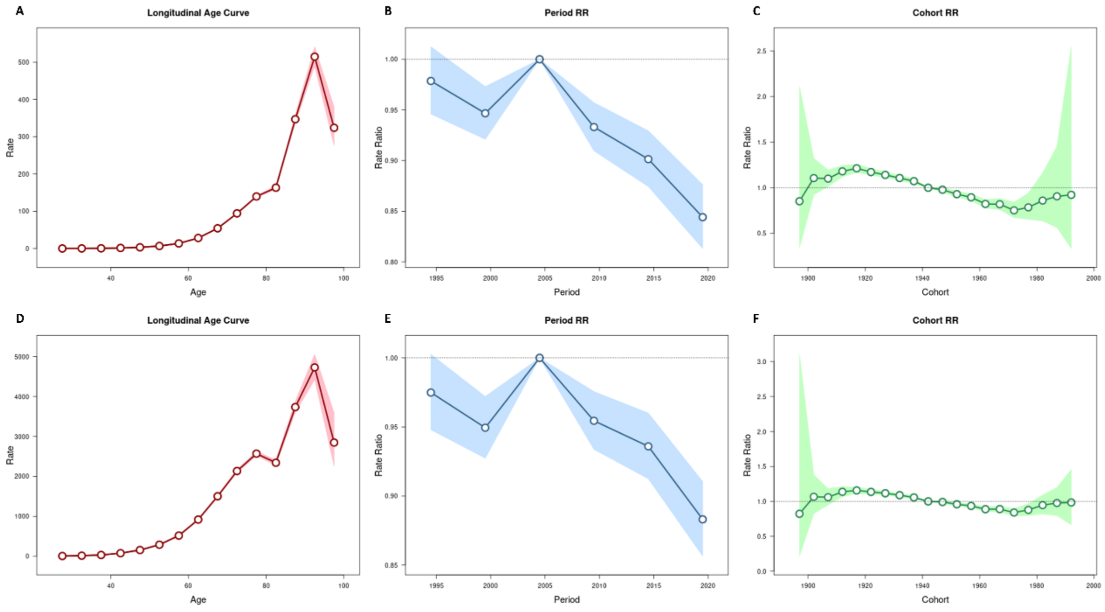

Supplementary FIGURE 2

Age–period–cohort analysis for death and DALY rates of ischemic stroke attributable to diet high in sodium in China among males. (A) Age efect for death rate. (B) Period efect for death rate. (C) Cohort efect for death rate. (D) Age efect for DALY rate. (E) Period efect for DALY rate. (F) Cohort efect for DALY rate.

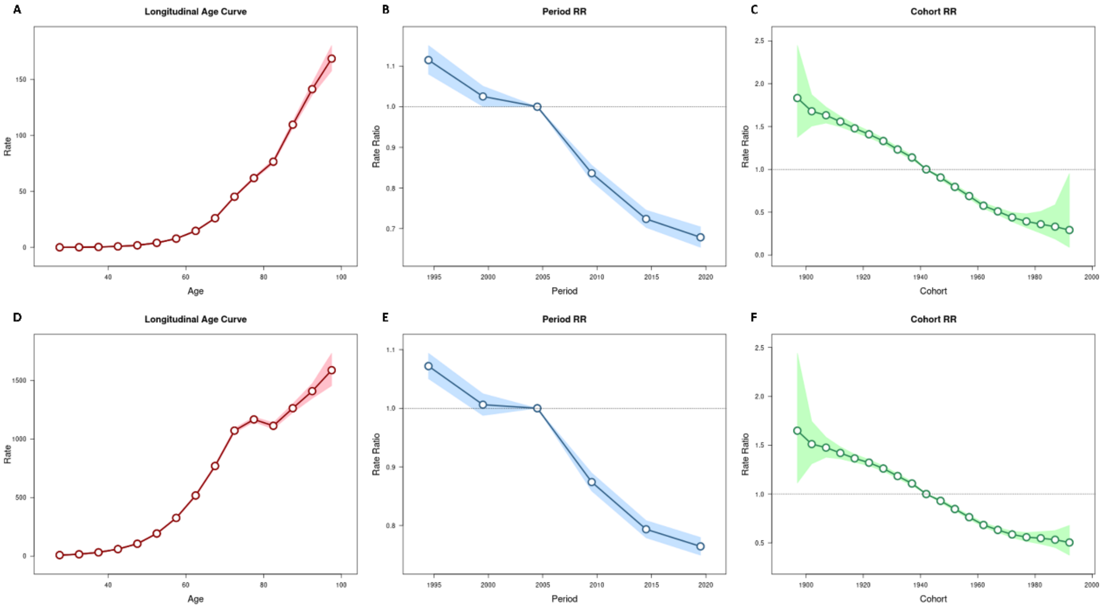

Supplementary FIGURE 3

Age–period–cohort analysis for death and DALY rates of ischemic stroke attributable to diet high in sodium in China among females. (A) Age efect for death rate. (B) Period efect for death rate. (C) Cohort efect for death rate. (D) Age efect for DALY rate. (E) Period efect for DALY rate. (F) Cohort efect for DALY rate.
