# Supplementary material for: Temporal trends of ischemic stroke attributable to diet high in sodium in China from the global burden of disease study 2021
Source: Front Nutr. 2025 Mar 13;12:1513981. doi: 10.3389/fnut.2025.1513981 (PMC11966442; doi:10.3389/fnut.2025.1513981)
Supplement: Supplementary file 3 [file Table_1.docx]

Supplementary Table 1

Burden of ischemic stroke attributable to diet high in sodium in China from 1990 to 2021

| Year | Number | | | | ASR, per 100,000 | | | |
| --- | --- | --- | --- | --- | --- | --- | --- | --- |
|  | Deaths | DALYs | YLDs | YLLs | Deaths | DALYs | YLDs | YLLs |
| 1990 | 76328 | 1902567 | 220601 | 1681966 | 11.43 | 241.79 | 25.58 | 216.21 |
| 1991 | 78212 | 1945786 | 229128 | 1716658 | 11.35 | 240.17 | 25.82 | 214.35 |
| 1992 | 80749 | 2002773 | 237769 | 1765004 | 11.37 | 240.37 | 26.07 | 214.31 |
| 1993 | 82646 | 2044273 | 245185 | 1799088 | 11.29 | 238.69 | 26.15 | 212.54 |
| 1994 | 83831 | 2069308 | 252740 | 1816569 | 11.13 | 235.16 | 26.23 | 208.93 |
| 1995 | 85781 | 2110815 | 259920 | 1850895 | 11.05 | 233.52 | 26.28 | 207.24 |
| 1996 | 87380 | 2142745 | 267289 | 1875456 | 10.94 | 230.85 | 26.31 | 204.54 |
| 1997 | 88465 | 2160476 | 274306 | 1886170 | 10.77 | 226.79 | 26.30 | 200.49 |
| 1998 | 90795 | 2206268 | 281186 | 1925083 | 10.74 | 225.57 | 26.26 | 199.31 |
| 1999 | 94227 | 2276854 | 288942 | 1987911 | 10.81 | 226.37 | 26.25 | 200.12 |
| 2000 | 99830 | 2387730 | 297052 | 2090679 | 11.15 | 231.55 | 26.29 | 205.26 |
| 2001 | 104500 | 2481628 | 306617 | 2175011 | 11.35 | 234.33 | 26.40 | 207.93 |
| 2002 | 107727 | 2558278 | 317484 | 2240793 | 11.32 | 234.37 | 26.60 | 207.76 |
| 2003 | 114391 | 2693661 | 330564 | 2363097 | 11.73 | 240.65 | 26.99 | 213.66 |
| 2004 | 120126 | 2816735 | 345079 | 2471655 | 11.95 | 244.48 | 27.39 | 217.09 |
| 2005 | 121181 | 2843246 | 359647 | 2483599 | 11.71 | 239.82 | 27.79 | 212.03 |
| 2006 | 116087 | 2766393 | 376798 | 2389594 | 10.77 | 224.56 | 28.26 | 196.29 |
| 2007 | 116193 | 2779548 | 396596 | 2382952 | 10.39 | 217.82 | 28.83 | 188.99 |
| 2008 | 120429 | 2867725 | 417201 | 2450524 | 10.38 | 217.51 | 29.39 | 188.12 |
| 2009 | 126060 | 2978769 | 439082 | 2539687 | 10.46 | 218.41 | 29.93 | 188.49 |
| 2010 | 131080 | 3080421 | 459445 | 2620976 | 10.48 | 218.51 | 30.34 | 188.16 |
| 2011 | 134436 | 3167843 | 479741 | 2688101 | 10.30 | 216.15 | 30.65 | 185.50 |
| 2012 | 136996 | 3245645 | 500920 | 2744725 | 10.02 | 212.69 | 30.96 | 181.74 |
| 2013 | 139685 | 3305188 | 522332 | 2782856 | 9.83 | 208.97 | 31.25 | 177.72 |
| 2014 | 142344 | 3364147 | 544752 | 2819395 | 9.61 | 204.78 | 31.48 | 173.30 |
| 2015 | 144711 | 3422721 | 565749 | 2856972 | 9.38 | 200.90 | 31.67 | 169.23 |
| 2016 | 150104 | 3534322 | 588018 | 2946304 | 9.33 | 199.83 | 31.84 | 167.99 |
| 2017 | 153971 | 3614954 | 610830 | 3004123 | 9.18 | 197.06 | 32.01 | 165.05 |
| 2018 | 157863 | 3698813 | 633512 | 3065301 | 9.04 | 194.48 | 32.15 | 162.34 |
| 2019 | 162749 | 3798643 | 656574 | 3142069 | 8.93 | 192.34 | 32.24 | 160.11 |
| 2020 | 168362 | 3909528 | 675898 | 3233630 | 8.88 | 191.26 | 32.18 | 159.08 |
| 2021 | 173363 | 4027007 | 713152 | 3313855 | 8.77 | 190.04 | 32.92 | 157.12 |

ASR, age-standardized rate; DALYs, disability-adjusted life years; YLDs, years of life lived with disability; YLLs, years of life lost.

Supplementary Table 2

Parameter estimates of age, period, and cohort effects on burden of ischemic stroke attributable to diet high in sodium in China among males.

| Type | Net drift (% per years; 95% CI) | P value | | |
| --- | --- | --- | --- | --- |
|  |  | All local  drifts=net drift | All cohort  deviations=0 | All period  deviations=0 |
| Death | -0.544(-0.763 to-0.352) | <0.001 | <0.001 | <0.001 |
| DALY | -0.333(-0.505 to-0.161) | 0.009 | 0.010 | <0.001 |

DALY, disability-adjusted life year

Supplementary Table 3

Parameter estimates of age, period, and cohort effects on burden of ischemic stroke attributable to diet high in sodium in China among females.

| Type | Net drift (% per years; 95% CI) | P value | | |
| --- | --- | --- | --- | --- |
|  |  | All local  drifts=net drift | All cohort  deviations=0 | All period  deviations=0 |
| Death | -2.095(-2.315 to-1.874) | <0.001 | <0.001 | <0.001 |
| DALY | -1.438(-1.533 to-1.344) | <0.001 | <0.001 | <0.001 |

DALY, disability-adjusted life year

Supplementary Table 4

The projected burden of ischemic stroke attributable to diet high in sodium by sex in China, 2022-2036.

| Year | Male | | Female | |
| --- | --- | --- | --- | --- |
|  | ASMR  (per 100,000) | ASDR  (per 100,000) | ASMR  (per 100,000) | ASDR  (per 100,000) |
| 2022 | 23.42 | 479.17 | 9.96 | 220.83 |
| 2023 | 23.21 | 475.20 | 9.79 | 218.35 |
| 2024 | 22.99 | 471.31 | 9.61 | 215.92 |
| 2025 | 22.78 | 467.53 | 9.44 | 213.53 |
| 2026 | 22.57 | 463.88 | 9.26 | 211.19 |
| 2027 | 22.35 | 460.36 | 9.09 | 208.92 |
| 2028 | 22.14 | 456.97 | 8.91 | 206.72 |
| 2029 | 21.94 | 453.69 | 8.74 | 204.59 |
| 2030 | 21.74 | 450.55 | 8.57 | 202.53 |
| 2031 | 21.55 | 447.59 | 8.40 | 200.57 |
| 2032 | 21.37 | 444.81 | 8.23 | 198.69 |
| 2033 | 21.19 | 442.20 | 8.07 | 196.90 |
| 2034 | 21.03 | 439.73 | 7.91 | 195.18 |
| 2035 | 20.87 | 437.41 | 7.75 | 193.55 |
| 2036 | 20.73 | 435.25 | 7.59 | 192.03 |

ASMR, age-standardized mortality rates; ASDR, age-standardized DALYs rates.
